# Supplementary material for: A fault-intrusion-tolerant system and deadline-aware algorithm for scheduling scientific workflow in the cloud
Source: PeerJ Comput Sci. 2021 Nov 2;7:e747. doi: 10.7717/peerj-cs.747 (PMC8576554; doi:10.7717/peerj-cs.747)
Supplement: Supplemental Information 6 [file peerj-cs-07-747-s006.zip › Myproject1.1/docs/org/cloudbus/cloudsim/provisioners/package-tree.html]

org.cloudbus.cloudsim.provisioners Class Hierarchy


JavaScript is disabled on your browser.


- Overview
- Package
- Class
- Use
- Tree
- Deprecated
- Index
- Help

- Prev
- Next

- Frames
- No Frames

- All Classes

# Hierarchy For Package org.cloudbus.cloudsim.provisioners

Package Hierarchies:

- All Packages

## Class Hierarchy

- java.lang.Object
  - org.cloudbus.cloudsim.provisioners.BwProvisioner
    - org.cloudbus.cloudsim.provisioners.BwProvisionerSimple
  - org.cloudbus.cloudsim.provisioners.PeProvisioner
    - org.cloudbus.cloudsim.provisioners.PeProvisionerSimple
  - org.cloudbus.cloudsim.provisioners.RamProvisioner
    - org.cloudbus.cloudsim.provisioners.RamProvisionerSimple

- Overview
- Package
- Class
- Use
- Tree
- Deprecated
- Index
- Help

- Prev
- Next

- Frames
- No Frames

- All Classes
